# Ageing of Goat Meat: Physicochemical Aspects, Sensory Changes and Quality Improvement Strategies

**DOI:** 10.3390/foods15183288

**Published:** 2026-09-17

**Authors:** Menalda V. André, Catarina Prista, Vítor D. Alves, Teresa J. S. Matos

**Affiliations:** 1 LEAF—Linking Landscape, Environment, Agriculture and Food Research Centre, Instituto Superior de Agronomia, Universidade de Lisboa, Tapada da Ajuda, 1349-017 Lisboa, Portugal; cprista@isa.ulisboa.pt (C.P.); matosteresa@isa.ulisboa.pt (T.J.S.M.); 2 Faculdade de Ciências de Saúde, Universidade Lúrio, Campus Universitário de Marrere, Nampula 4250, Mozambique; 3 Associate Laboratory TERRA, Instituto Superior de Agronomia, Universidade de Lisboa, Tapada da Ajuda, 1349-017 Lisboa, Portugal

**Keywords:** aged meat, flavor characteristics, meat quality, microbiological safety, tenderness

## Abstract

Goat meat ageing is a promising strategy for improving meat quality and adding value to meat from adult animals. This review aimed to provide an integrated overview of goat meat ageing, focusing on biochemical, technological, physicochemical, sensory and microbiological aspects. A literature search was conducted in Scopus, Web of Science and ScienceDirect using combinations of terms related to goat meat, ageing technologies, meat quality and microbial quality. Relevant studies were selected through title, abstract and full-text assessment. Evidence from goat meat was prioritized, while studies on sheep and beef were used as supplementary evidence when goat-specific information was limited. The reviewed evidence indicates that ageing can improve goat tenderness and flavor through *post-mortem* proteolysis and associated biochemical changes. Dry ageing generally promotes greater flavor development, whereas wet ageing offers advantages for moisture retention and yield. Dry ageing in bags represents a promising alternative by potentially combining sensory benefits with reduced losses. However, ageing responses vary according to muscle type, slaughter age, ageing time, temperatures and packaging system, while microbiological safety remains an important consideration. Overall, further research is needed to establish goat-specific ageing conditions and integrate microbiological, physicochemical, sensory, consumer, and economic assessments to support the development of safe, acceptable and commercially viable aged goat meat products.

## 1. Introduction

Small ruminants play an important role in meeting the growing global demand for meat due to their chemical and physical composition, nutritional properties and distinctive sensory characteristics [1]. Goat meat is recognized worldwide for its high nutritional quality and is considered one of the leanest and healthiest red meat options [2]. Historically, and across various cultures, goat meat has been an essential source of nutrients; it is also free from religious taboos, which contributes to its widespread acceptance in many regions of the world [3]. This product contains high levels of protein, B vitamins, essential fatty acids, and various minerals essential to human health [4], as well as a higher proportion of polyunsaturated fatty acids than that of other ruminants [5,6]. However, the characteristic flavor of goat meat may influence consumer acceptance [4]. The sensory variation often observed among goat products results in distinct taste experiences that can elicit both positive and negative perceptions [1]. The sensory profile of this meat arises mainly from complex interactions among components such as fat, proteins, amino acids and small volatile compounds [4].

In addition, fatty acids, particularly oleic and linoleic acids, influence the formation of volatile compounds responsible for the characteristic aroma of goat meat [7]. It should also be noted that the meat of older animals exhibits lower tenderness, which has been associated with greater collagen cross-linking in connective tissue [8,9]. In addition, meat from older goats tends to have a lower intramuscular and subcutaneous fat content, resulting in a leaner and firmer product when compared with beef and sheep meat, contributing to a lower consumer preference [10,11].

Sustainable meat production is the key to mitigating the environmental impacts of the livestock industry [2]. In this context, goat farming emerges as a valuable alternative, playing a decisive role in promoting agricultural sustainability, strengthening rural economic dynamics and preserving the ecosystem. Goat farming contributes significantly to the diversification of agricultural holdings, promotes the socioeconomic resilience of local communities, and encourages the adoption of environmentally responsible practices through the efficient management of natural resources [3] and the enhancement of marginalized territories. Goats, as multifunctional species, represent an underutilized source of red meat, with characteristics particularly well suited to sustainable production [2]. In addition, they are highly adaptable to harsh environments, capable of withstanding extreme weather conditions, long periods of water deprivation and limited land conditions [2,12,13,14].

Goats are distinguished by their diverse diet and high adaptive capacity, feeding on both grasses and shrub species typically avoided by other ruminants, making them ideal for diversified and sustainable production systems [2]. At the same time, goat production systems are characterized by the efficient use of water and energy resources and reduced dependence on non-renewable energy sources, which contributes to a reduction in the environmental footprint of production systems based on this species [15]. In addition, goats emit less methane than do other domestic ruminants [16]. The digestive system of goats is highly efficient at converting low-quality fodder and agricultural waste into high-nutritional-value food products and produces manure that positively impacts soil fertility and health [13]. This feed efficiency allows goat farming to be carried out with a reduced requirement for external inputs, often eliminating the need for vitamin supplements [2]. In addition to these feeding abilities, goats show remarkable physiological robustness, reflected in their ability to maintain reproduction and productivity even under adverse environmental conditions [17]. This robustness stems from homeostatic regulatory mechanisms that maintain internal balance and homeorhetic responses that regulate metabolic tissues according to reproductive needs. This performance can be optimized through genetic selection, nutritional management and the promotion of animal welfare, which are essential factors for sustaining productive stability in environments subject to climatic or food limitations [17]. Thus, goat farming plays a strategic role in the current context of climate change and evolving consumer preferences, presenting itself as a sustainable alternative to red meat production that promotes the conservation of natural resources, agricultural biodiversity, and the development of rural communities [2].

Meat ageing is a complex post-mortem process that enhances the palatability of meat products, making them more appealing to the human palate. This process, triggered by endogenous proteases [18,19,20], is characterized by a series of biochemical and physicochemical transformations that significantly improve the sensory quality of meat by increasing tenderness and developing unique flavors over time [19,20,21,22,23]. During ageing, proteolysis and lipolysis break down large, tasteless molecules into smaller, flavorful fragments, resulting in changes in the lipids, organic acids, vitamins, and nucleotide-bound phosphates of meat [24]. These degradation products contribute to the intense meat flavor and nutty notes of aged meat [25].

Consumers generally prefer meat from young animals because they perceive it as more tender [5,26]. In this context, the advanced age of animals influences the quality attributes of meat, particularly texture and color, thereby limiting its commercial value [5]. However, meat ageing has emerged as a technological strategy that improves the tenderness and sensory properties of meat from older animals, thereby enhancing its commercial value in premium red meat markets [27]. Although primarily established in the beef sector, beef processors are revalorizing traditional ageing methods, recognizing them as an effective way to improve product quality and to achieve market differentiation [28].

The aim of the present review is to examine the effects of ageing on goat meat quality, with a particular emphasis on the physicochemical, sensory and microbiological changes occurring during the process. The review also discusses the main factors influencing these changes and their implications for key quality attributes, i.e., tenderness, juiciness, flavor, color and microbiological quality. By integrating these aspects, this review provides an updated perspective on ageing as a strategy for improving and valorizing goat meat quality.

## 2. Methods

In this review, a comprehensive methodology was adopted to explore aspects associated with the meat ageing process. Studies focusing on the application of meat ageing techniques were considered, with an emphasis on their effects on food safety, physicochemical properties and quality parameters, particularly texture and sensory characteristics.

To gather a broad spectrum of relevant literature, a search was conducted across multiple scientific databases, including Scopus, Web of Science and ScienceDirect. No time limit was applied to the literature search, and relevant publications retrieved up to the final search date, 6 February 2026, were considered. In addition, the reference lists of the selected articles were consulted with the aim of identifying relevant studies that had not been retrieved through the database search.

The search strategy was based on a combination of keywords and Boolean operators. The main keywords used included: “goat meat”, “chevon”, “ageing”, “dry ageing”, “dry ageing in bag”, “wet ageing”, “meat quality”, and “microbial quality”. The terms were combined using the Boolean operators “AND” and “OR”. A representative search string was: (“goat meat” OR “chevon”) AND (“ageing” OR “dry aging” OR “dry ageing in bag” OR “wet ageing”). Search terms were adapted as necessary to the specific requirements of each database.

The retrieved records were initially selected based on their titles and abstracts, followed by an assessment of the full text to evaluate their relevance and consistency with the scope of this review. Studies addressing ageing techniques applied to goat, sheep and beef meat, as well as pork, where relevant, were included as supplementary evidence, particularly when they provided relevant information on biochemical mechanisms, physicochemical changes, sensory characteristics, microbiological quality or ageing conditions potentially applicable to goat meat. However, the results obtained from different species were considered separately, and their direct applicability to goat meat was not assumed.

Studies focusing on the ageing of fish, horse meat, and rabbit meat were excluded, as were those focusing exclusively on other products of caprine origin, namely milk and dairy products. Publications addressing aspects unrelated to meat quality or ageing were also excluded, as were those whose information was not relevant to the objectives of this review.

The selected literature was subsequently analyzed and organized according to the main themes addressed in this review, namely physicochemical changes, sensory characteristics, the biochemical and microbiological mechanisms associated with ageing, and strategies used to improve the quality of goat meat. Throughout the analysis, evidence obtained directly from goat meat was distinguished from that derived from other species, with the latter being used as supplementary support when specific evidence for goats was limited.

## 3. Biochemical Mechanisms of the Meat Ageing Process

Meat quality results from multiple biochemical processes that occur in muscle tissue after slaughter. Among these, glycolysis stands out because it directly influences muscle pH and consequently, characteristics such as color, texture, and juiciness [29]. Differences in the rate and extent of *post-mortem* glycolysis have been associated with the development of pale, soft, and exudative (PSE), as well as dark, firm, and dry (DFD) characteristics in meat [29]. Myofibrils, composed mainly of myosin, play a central role in the texture, water-holding capacity and sensory attributes of meat [30]. Thus, *post-mortem* changes in myofibrillar structure may influence the quality characteristics of goat meat [30].

Texture is one of the attributes most valued by consumers, directly influencing sensory acceptance and perception of product quality [31]. Among the parameters that define it, tenderness stands out as a determining characteristic, being strongly associated with the palatability and juiciness of the final product [31,32]. Ageing, as a post-mortem intervention widely used in the meat industry, significantly enhances meat tenderness through the natural proteolysis of structural proteins [33].

During the *post-mortem* period, muscle tissue undergoes profound structural changes. These transformations are modulated by physiological and environmental factors before slaughter, which determine the subsequent biochemical response. In addition to the intrinsic mechanisms of muscle contraction and subsequent relaxation, *ante-mortem* factors, such as the animal’s diet and metabolic state, exert a decisive influence on the biochemical phenomena that follow slaughter. Diet composition can alter the proportions of muscle energy reserves, thereby affecting pH decline and carcass cooling rate, which are essential parameters for determining meat tenderness and juiciness [11]. In addition, the composition and structure of myofibrillar proteins, influenced by the type and energy density of the diet, affect the proteolytic response during ageing [11]. Studies indicate that meat from animals fed low-energy diets tends to have better texture than that from animals fed high-energy diets. However, goat meat (chevon) is characterized by the higher collagen levels and more stable connective tissues throughout ageing [11], which limits the extent of tenderization achievable through post-mortem proteolysis.

The rate and extent of meat tenderization vary and depend not only on *ante-mortem* factors but also on intrinsic factors such as animal species, age, and predominant muscle fiber type, as well as extrinsic factors including temperature, storage type, and the duration of the ageing period [34]. These factors influence the intensity of myofibrillar proteolysis and partly explain the variability observed in the response to ageing between different species, including goats, and production systems. 

*Rigor mortis* begins with the interruption of cellular metabolism and the depletion of adenosine triphosphate (ATP) reserves, leading to the formation of cross-bridges between actin and myosin filaments [31]. In bovine muscle, this phenomenon is complete approximately 24 h after slaughter. *Rigor mortis* develops progressively at the level of individual muscle fibers, culminating in the irreversible association between thick and thin filaments after ATP depletion, a process influenced by the initial level of glycogen present in muscle cells before slaughter [34]. In lamb studies, it has been reported that tenderization begins only after *rigor mortis* resolution, although proteolytic activity can commence before the entire muscle reaches *rigor mortis* [35]. After *rigor mortis* resolution, and throughout aging, meat undergoes complex biochemical changes resulting from the proteolytic activity of enzymes such as calpains, cathepsins, and caspases, which degrade myofibrillar and cytoskeletal proteins [31,33]. Research in lamb has shown that, although low temperatures slow this activity, they do not completely inhibit it, allowing proteolysis to continue gradually [36]. This degradation promotes the fragmentation of muscle fibers and consequently, the progressive tenderization of the meat [33].

At the same time, the combined action of proteolytic and lipolytic enzymes promotes the formation of flavor precursor compounds, such as small peptides, free amino acids, and fatty acids, which determine the characteristic profile of aged meat [32]. Based on studies with beef, among the amino acids released, asparagine and glutamine are associated with sweet notes, while isoleucine, leucine, phenylalanine, tyrosine, and valine impart bitter and slightly sweet flavors, and methionine contributes to the umami flavor typical of cooked meats [37]. It is important to note that the amino acid profile and lipid composition differ between species, so flavor development in goat meat may present characteristics distinct from those described for beef. Additionally, in studies on pork, it has been reported that during vacuum-packaged ageing, fermentative reactions driven by lactic acid bacterial metabolism may occur, leading to ethanol formation [38]. Although this phenomenon has not been specifically reported in goat meat, it is plausible that similar reactions may occur, depending on packaging and temperature conditions.

Although several endogenous proteolytic systems can promote protein degradation, the calpain system is widely recognized as the primary system responsible for improving tenderness during ageing [33]. Among the enzymes, calpain-1 (μ-calpain) and calpain-2 (m-calpain) isoforms stand out as the main proteases involved in the degradation of myofibrillar proteins [38]. In bovine muscle studies, calpain-1 exhibits high activity in the early stages of *post-mortem* aging, while calpain-2, previously considered inactive due to acidic conditions and low calcium concentration in the muscle, proves to be functional in more advanced stages, especially after 14 days of ageing [39,40].

Tenderization results from the synergistic action of calpains and cathepsins, which catalyze the gradual degradation of myofibrillar and cytoskeletal structural proteins [33,41]. Although other enzymes, such as caspases, contribute to muscle proteolysis, the role of calpains remains predominant [39]. Among the main target proteins are desmin, tropomyosin, titin, nebulin, and troponin T, whose degradation compromises sarcomere integrity, leading to myofibril disintegration and the consequent loss of myofibrillar integrity [33]. In pork studies, desmin has been identified as a cytoskeletal protein of high structural importance, and its degradation is considered one of the first indicators of muscle proteolysis [38,42]. Based on investigations in beef, desmin is a substrate of calpain-2, whose enzymatic activity contributes to the fragmentation of muscle structures in more advanced stages of ageing [43]. This degradation process has direct implications for improving water-holding capacity and reducing meat shear force [38,42]. As calpains degrade muscle structure, desmin is often used as a biochemical marker to evaluate proteolysis and meat tenderization, with its utility validated across multiple beef *in vitro* studies [42].

## 4. Types of Ageing Processes and Technological Parameters

The ageing process is carried out by subjecting whole carcasses, primary, or sub-primary cuts, particularly ribs and loins, to controlled temperature, relative humidity, and air-circulation speed conditions [24,25,44,45]. These conditions aim to promote natural proteolysis without compromising microbiological safety. The use of ultraviolet (UV) light has also been reported as effective in eliminating airborne microorganisms, contributing to the hygienic stability of the ageing environment [25].

### 4.1. Ageing Process

**Figure 1:**
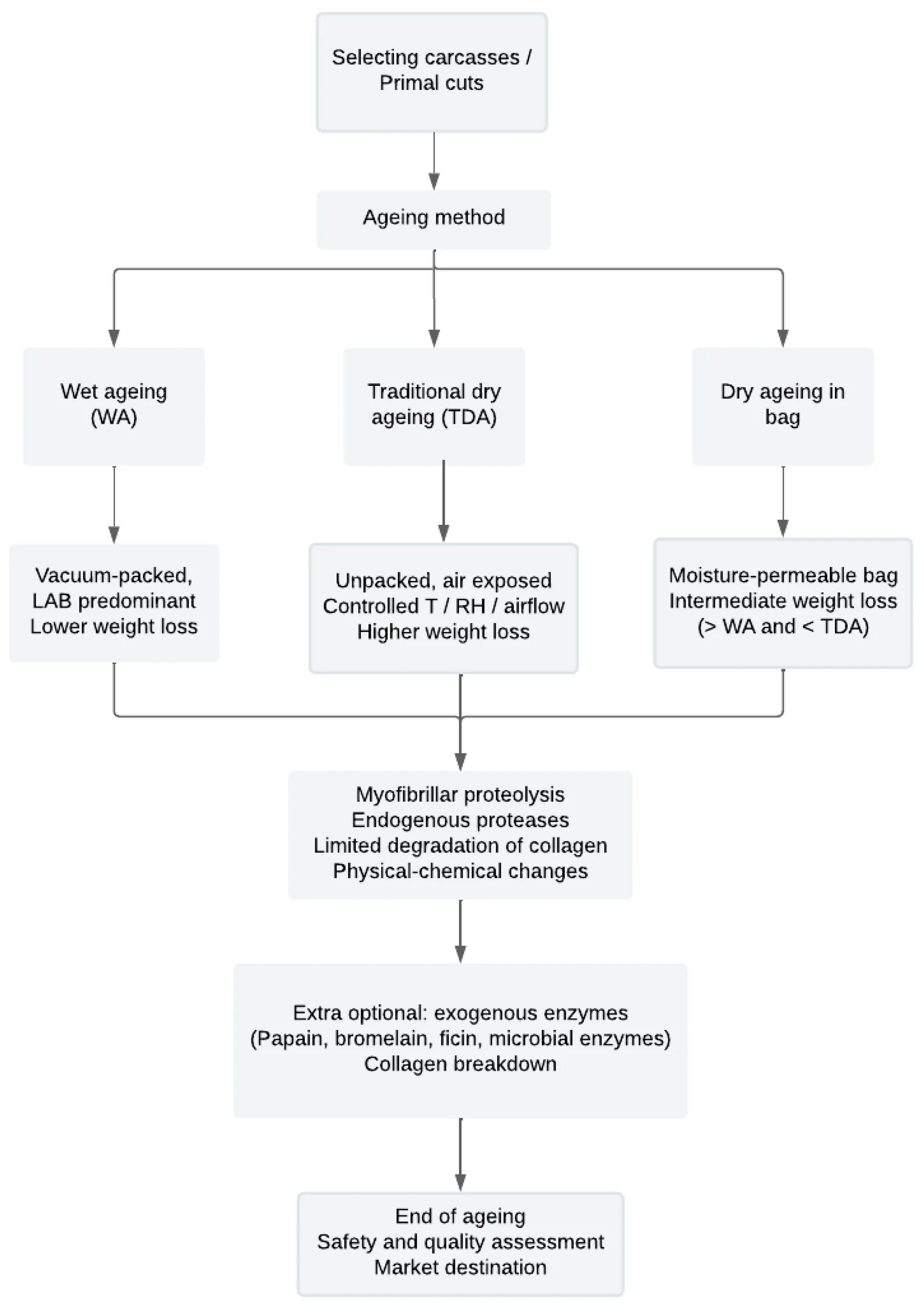
Flowchart illustrating meat ageing methods (wet, traditional dry, and dry ageing in a bag), highlighting the main biochemical changes and complementary strategies through the use of exogenous enzymes (T: temperature, RH: relative humidity, and LAB: lactic acid bacteria).

Essentially, two aging techniques dominate modern meat production: dry ageing and wet or vacuum ageing [46,47,48]. For centuries, dry ageing was a common practice among butchers to preserve and tenderize meat, and it remained the norm until about 50 years ago [49]. However, with the advent of vacuum packaging and increased efficiency in meat processing and transport, this method lost popularity. Before the 1970s, dry ageing of non-packaged carcasses and cuts in cold storage represented the primary preservation method. With the development of vacuum packaging techniques, wet ageing became increasingly widespread, supplanting dry ageing [23,49]. The wet ageing approach (Figure 1) became the predominant ageing method due to its low weight loss and convenience during storage and transport [50]. This technique consists of anaerobic ageing of meat, carried out in vacuum barrier packaging and stored under refrigeration for a period ranging from one to several weeks, during which endogenous enzymes act on the muscle tissue, promoting its tenderization, albeit with the formation of new compounds responsible for flavor development being limited [18,47,48]. The meat industry considers it the most practical ageing method due to its minimal processing requirements and negligible yield loss [24]. In addition, meat subjected to wet ageing has a moderate flavor characterized by metallic notes, distinguishing it from dry-aged meat [51].

On the other hand, dry ageing (Figure 1) consists of storing meat under controlled temperature and humidity conditions, without packaging, allowing surface moisture to evaporate and flavor compounds to concentrate [46]. At the same time, natural microbial activity contributes to the development of complex aromas, including buttery, earthy, and umami notes [46]. Recently, there has been growing consumer interest in dry-aged meat, which has been served more frequently in luxury hotels and restaurants, where the ageing process is often carried out in refrigerated display cases visible to the public [28]. However, this technique is used only by a limited number of suppliers serving luxury restaurants and specialized (gourmet) markets [24].

Exposing the meat surface to the ageing environment promotes an aerobic process responsible for a unique and intensified flavor profile, characterized by notes of dried fruit, meat, and roasted and earthy flavors. These meat products are considered superior in quality and consequently, command a higher commercial value than their wet-aged counterparts. However, dry ageing incurs additional costs due to weight loss from dehydration, the inevitable trimming of scraps, reduced marketable yield, increased risk of microbial contamination, and additional infrastructure requirements for ageing [24]. These limitations make the process particularly costly, directly affecting the final price of dry-aged meat [28].

Conventionally recommended for about three weeks, this process stimulates endogenous proteases that are efficient at degrading myofibrillar proteins but have limited ability to hydrolyze native collagen, the main component of connective tissue responsible for firmness in meat from older animals [10]. Consequently, although ageing improves tenderness through myofibrillar proteolysis, it is insufficient to effectively reduce the toughness associated with stabilized collagen in meat from older animals. This limitation justifies the need to resort to appropriate tenderizing processes using various technological interventions to degrade both connective tissue and myofibrillar proteins [52]. This constraint, together with the high energy costs and increased space requirements associated with prolonged ageing, has driven the development of alternative strategies that apply exogenous enzymes (Figure 1). Consequently, the application of exogenous proteases or combined ageing strategies may offer greater benefits for improving the tenderness of goat meat, warranting further investigation in goat-specific studies.

Exogenous proteases, of plant and microbial origin, are distinguished by their ability to act not only on myofibrillar proteins, but also on connective tissue components, promoting changes in muscle microstructure and consequently, increasing meat tenderness [53]. Among the most studied are papain, bromelain, ficin, and actinidin, as well as microbial proteases from species such as *Aspergillus oryzae* and *Bacillus subtilis*, which are classified as “generally recognized as safe” by the USDA Food Safety Inspection Service [9,10,53,54]. However, the application of these enzymes requires strict control of the conditions of use, since their broad substrate specificity can lead to excessive hydrolysis of muscle proteins, resulting in undesirable changes in the texture and sensory profile of meat [10,53].

In this context, alongside approaches based solely on exogenous enzymes, the exploration of combined dry and wet ageing schemes, recently described in the literature [55,56], has gained importance. In general, this combination offers a favorable balance between water loss, flavor development, and yield retention, with the potential to enhance the overall quality of the final product [55]. Additionally, to address the low yield, lipid oxidation, and microbial contamination associated with dry ageing, an innovative method, dry ageing in bags (Figure 1), was developed [55,57]. This method uses packaging with high water vapor permeability, allowing moisture to evaporate during the ageing process while also serving as a protective barrier that limits access of oxygen and microorganisms from the surrounding environment [57]. Moisture-permeable packaging is typically made of polymers such as thermoplastic urethane, which are known for their softness, elasticity, good cold resistance, and anti-mold properties, all of which help mimic dry ageing conditions while preserving product integrity [58].

Dry ageing in bags provides superior performance compared with traditional dry ageing, reducing losses associated with excessive trimming and microbial contamination without compromising meat tenderness and sensory quality. Despite the slight increase in costs associated with bag use, the improvement in the meat sensory profile achieved through dry ageing in bags offers considerable potential to increase economic returns for the meat industry by enabling the development of new premium products targeting high-value markets [24].

### 4.2. Effect of Ageing Time

The duration of the ageing period is one of the main factors determining the sensory quality of meat, directly influencing its flavor intensity and complexity. Extending this period allows greater enzymatic activity, favoring the formation of compounds that enrich the product’s sensory profile [46]. However, this process must be carefully controlled, as excessive ageing can also increase the risk of meat deterioration [46]. Several studies involving beef have shown that ageing time significantly impacts tenderness, juiciness, and overall meat quality, contributing to the development of compounds that intensify flavor and aroma [18]. In general, intervals between 14 and 40 days are often reported as effective for beef to achieve an adequate balance among tenderness, flavor, and juiciness [22,25,44,49]. Metabolomic studies of beef also indicate that around 21 and 28 days of ageing, there is a marked accumulation of small peptides, free amino acids, and their derivatives, reinforcing the role of this interval as a critical window for the development of desirable sensory attributes [47].

Despite these positive effects, there is evidence that prolonged ageing does not always translate into additional improvements in tenderness or sensory perception. Some studies report that prolonged refrigerated storage does not yield additional gains in shear force or sensory evaluation, suggesting that optimal time limits vary according to species, muscle type and storage conditions [59]. Based on these results, shorter ageing periods have been recommended, specifically 7 to 10 days for lamb meat at temperatures of 1 and 4 °C [60], about 2 weeks for goat meat aged in a humid environment at 2 °C [27], and about 2 to 3 weeks for beef kept at temperatures of 1 and 2 °C [59]. However, other studies indicate that the optimal tenderness of beef can only be achieved after substantially longer periods, up to about 12 weeks, when stored at −1 °C, suggesting that the traditionally proposed limits should be re-evaluated, particularly considering the role of storage temperature [59,61].

In fact, it has been shown that the tenderizing process can take longer when beef is aged at lower temperatures (e.g., around 0 °C). At the same time, in lamb meat, optimal tenderness can be achieved more quickly when higher storage temperatures (3 to 7 °C) are used, compared to the results for longer ageing at low temperatures. These differences have been associated with increased calpain-1 activity at moderately higher temperatures, highlighting the critical interaction between time and temperature in controlling proteolysis. It should be noted that meat tenderness depends not only on the ageing time but also on the type of muscle and the composition of the connective tissue comprising each cut [62]. Consequently, different muscles require different ageing periods to achieve the conditions considered ideal by consumers [62]. From this perspective, the optimal time window can be adjusted by approximately ±7 days, depending on the breed and degree of marbling, with longer dry ageing periods (45–60 days) typically reserved for beef carcasses with high marbling, such as Wagyu, in which the intramuscular fat content contributes to reducing moisture loss and intensifying complex flavor notes [47].

On the other hand, studies using beef have demonstrated that shorter ageing periods, between 7 and 14 days, may be sufficient to improve tenderness and selected sensory attributes [21], although the magnitude and nature of these effects depend on the muscle type, ageing conditions and evaluation criteria. Longer ageing periods may further modify the sensory profile through the accumulation of volatile compounds and other metabolites, potentially resulting in more intense or complex aroma and flavor characteristics. However, evidence regarding ageing periods exceeding 40 days remains limited, particularly for beef, and the potential benefits of such prolonged ageing must be considered alongside possible drawbacks, including oxidative deterioration, undesirable flavor development, color changes and increased processing losses. In goat meat, the available evidence is insufficient to establish a generally applicable optimal ageing duration, highlighting the need for controlled studies under clearly defined experimental conditions. Therefore, ageing time should not be regarded as a universal parameter, but rather as a factor whose effects depend on the interaction among species, muscle type, animal age at slaughter, packaging system, storage temperature and the specific quality attribute under evaluation.

### 4.3. Ageing Temperature

Regarding the environmental conditions during ageing, temperature is a critical factor influencing the rate of biochemical reactions, microbial growth and consequently, the progression and safety of the ageing process. The appropriate ageing temperature should be established in conjunction with other environmental and technological parameters, including relative humidity, air velocity, ageing time, animal species, muscle type, slaughter age, packaging system and the quality attributes being targeted. For dry aged beef, European legislation [63] establishes specific conditions under which the process may be carried out, including a surface temperature between-0.5 and 3.0 °C, a maximum relative humidity of 85%, and air velocity of 0.2–0.5 m/s for a maximum of 35 days. However, the legislation also recognizes that alternative combinations of these parameters, as well as the dry ageing of meat from other species, may be applied, provided that equivalent guarantees of meat safety are demonstrated [63].

In studies involving goat meat, ageing temperatures have generally ranged from 0 to 4 °C [5,27,57,62], as summarized in Table 1 and Table 2. These temperatures are particularly relevant because temperatures close to the freezing point may reduce the rate of the enzymatic processes responsible for key biochemical transformations [64]. Conversely, temperatures above this range favor microbial proliferation, accelerating deterioration phenomena that manifest as unpleasant odors, loss of flavor, and the possible development of pathogenic microorganisms [49,64].

### 4.4. Relative Humidity

Relative humidity is a critical parameter in the dry ageing process of meat, directly influencing its microbiological quality, physicochemical stability, and sensory attributes. Although it is repeatedly cited in the literature as one of the most relevant factors in this process, there is no absolute consensus on the ideal value to use [64]. Studies report wide variation in relative humidity levels, ranging from 49% to 98%, often without rigorous measurements or precise control of environmental conditions [67], making it difficult to draw consistent conclusions. The literature generally recommends a range of 80% to 85% [25], with 80% often cited as the reference [64]. However, different studies show that variations in relative humidity can influence flavor development. For example, Kim et al. [68] observed numerically higher sensory scores in dry-aged meat at 49% relative humidity, while [69] found greater acceptability when relative humidity was gradually increased from 65–75% to 85%. Similarly, Vossen et al. [64] reported that higher relative humidity (90%) resulted in lower typical flavor ratings than those observed at 70%. These results suggest that relative humidity plays a decisive role in the formation of the characteristic flavor notes of dry ageing. From a technological point of view, high relative humidity favors mold growth, while excessively low levels can cause excessive drying of the meat, compromising its juiciness [25].

### 4.5. Air Velocity

Air velocity is a determining parameter in the dry ageing process of meat, directly influencing the microbiological and physicochemical characteristics of the product’s surface. Different air velocities significantly alter the microbial composition in the crust of dry-aged beef, leading to variations in the pH and compounds responsible for flavor development [70]. However, it has also been observed that changes in air velocity, when considered in conjunction with other processing factors, do not necessarily result in significant differences in the final pH of the meat, suggesting that the effect of this parameter depends on experimental conditions and interactions among environmental elements such as temperature and relative humidity [68]. At the same time, air velocity plays an essential role in promoting the surface drying of meat, helping to limit the growth of undesirable microorganisms, such as bacteria and molds [25]. More specifically, speeds generally ranging from 0.2 to 0.5 m/s are considered adequate to ensure uniform surface drying, preventing the formation of moisture-accumulating areas that could serve as niches for spoilage bacteria [71].

## 5. Microbiological Safety of Aged Meat

Meat is a highly perishable product due to its intrinsic properties, particularly its high water content and water activity very close to 1 (a_W_ > 0.99), as well as its pH, generally between 5.5 and 6.5, which is conducive to microbial growth [72]. These characteristics are associated with the abundant availability of nutrients such as amino acids, nucleosides, vitamins, and minerals, which provide metabolic conditions conducive to the growth of microorganisms. These conditions render meat a substrate particularly susceptible to biological deterioration, which justifies its limited shelf life [72].

According to EFSA opinion, meat spoilage is primarily caused by *Pseudomonas* spp., *Lactobacillus* spp., *Enterococcus* spp., *Weissella* spp. and *Brochothrix* spp. (producers of viscous exudate) and *Shewanella* spp. and *Clostridium* spp. (causers of color changes), with the development of characteristics odors such as sulfide, cabbage and souring. Under aerobic conditions (dry ageing), *Pseudomonas* spp. predominate, while under anaerobic conditions (wet ageing), lactic acid bacteria (LAB) and *Clostridium* spp. are the main spoilage microorganisms. The main microbiological hazards associated with aged meat include Shiga toxin-producing *Escherichia coli*, *Salmonella* spp., *Staphylococcus aureus*, *Listeria monocytogenes*, *Yersinia enterocolitica*, *Campylobacter* spp. and *Clostridium* spp. [73].

In addition to these spoilage microorganisms, characteristic molds and yeasts have been identified in aged meat, which simultaneously influence the product’s sensory quality and microbiological safety [48]. Molds, including species of the genera *Aspergillus* spp. and *Penicillium* spp., may produce mycotoxins under favorable environmental conditions. Their development can be controlled by maintaining the meat surface at −0.5 to 3.0 °C, with relative humidity between 75 and 85% and an airflow of 0.2–0.5 m/s for a maximum of 35 days [73].

In the European context, Regulation (EU) 2024/1141 [63], amending Regulation (EC) No. 853/2004 [74], introduces specific provisions for the dry ageing of meat. The regulation defines the dry-ageing process and establishes specific conditions regarding temperature, relative humidity and airflow, as well as maximum ageing time, to be followed by food business operators. At the same time, Regulation (EC) No. 2073/2005 [75] establishes microbiological criteria for specific food categories and microorganisms, including criteria for certain ready-to-eat foods concerning pathogens such as *Listeria monocytogenes* and *Salmonella* spp. and provides the regulatory framework for microbiological safety. In this context, the control of dry ageing conditions requires appropriate monitoring of temperature, relative humidity and airflow, as well as calibration and maintenance records for the equipment [47], to ensure effective control of process conditions and compliance with current legislation.

In the context of ageing, several studies indicate that initial slaughter and handling conditions can have a significant influence on the initial microbial load [76,77]. Ensuring that aged meat meets microbiological safety standards is essential to prevent foodborne diseases and maintain product quality and consumer confidence [36]. Safety parameters are usually assessed by counting the total number of aerobic microorganisms [78], which is recognized as one of the most relevant indicators of meat hygiene and sanitary quality [79]. However, during the ageing process, the total bacterial load and spoilage microorganisms can reach 5–6 Log_10_ CFU/g without visible signs of change, with bacteria of the genus *Pseudomonas* frequently dominating the meat surface [80].

Studies indicate that levels of 6–8 Log_10_ CFU/g of psychrophilic microorganisms are sufficient to cause unpleasant odors, surface viscosity, and appearance defects, while total counts close to 7 Log_10_ CFU/cm^2^ or g are associated with typical flavors of deterioration, although sensory changes may occur between 5–6 Log_10_ CFU/cm^2^ [55]. This fact limits the isolated use of total mesophilic or psychrophilic counts as the sole indicator of hygienic conditions in aged meat [80]. Ageing under defined and controlled conditions can achieve the same or lower loads of microbiological hazards and spoilage bacteria compared to the levels for standard fresh meat preparation [73].

In small ruminants such as sheep and goats, the microbial load is generally higher than that observed in cattle due to factors such as animal hygiene conditions and processing practices [81]. In wet-aged goat meat (Table 2), the total aerobic count evolved from initial values close to 3 Log_10_ CFU/g to around 6.5 Log_10_ CFU/g after 14 days of ageing, remaining acceptable, and exceeding 7 Log_10_ CFU/g at 21 days, a value considered to be the threshold for sensory and microbiological deterioration [10].

The microbiological safety of aged goat meat must be ensured in accordance with the principles of the Hazard Analysis and Critical Control Point (HACCP) system, in conjunction with the program of prerequisite conditions and good manufacturing practices [73,80]. Strict implementation of these programs at all stages of the production chain, including the ageing process, is essential to preserve the safety and quality of the final product [80].

## 6. Characteristics of Aged Goat Meat

Goat meat is recognized for its nutritional characteristics and its relevance as a sustainable red meat source owing to the adaptability of goats to diverse production environments [2,3,82,83,84,85]. In this context, ageing represents an important *post-mortem* strategy for improving goat meat quality. During ageing, endogenous enzymatic processes contribute to structural changes in muscle, promoting improvements in tenderness and influencing other quality attributes, including flavor [3].

However, factors such as pre-slaughter stress can inhibit the activity of these enzymes, thereby slowing proteolysis and compromising quality parameters during ageing [86]. The extent of these changes is also influenced by *post-mortem* conditions, including ageing temperature, duration, and storage conditions. Thus, proper control of the ageing process, together with appropriate management of pre-slaughter factors and *post-mortem* conditions, is crucial for obtaining meat with optimized texture, flavor, color and pH (Table 1).

Forte et al. [27] reported that dry and wet ageing affected several quality attributes of goat meat, including tenderness, water-holding capacity, cooking loss, and color, although the magnitude and timing of these changes differed between ageing systems. Dry-aged goat meat showed higher water-holding capacity and lower cooking loss than wet-aged meat, while WBSF decreased during the first and the second week of dry ageing, indicating an improvement in instrumental tenderness. Dry ageing also promoted changes in volatile compounds and sensory attributes, with the first two weeks associated with increased tenderness and improved flavor, whereas prolonged ageing was associated with a decline in overall quality [27].

Similarly, Ali et al. [62] reported that the effects of wet ageing on goat meat varied according to muscle type and ageing duration. Five days of ageing improved tenderness in *Longissimus lumborum*, while in *Biceps femoris*, it increased water-holding capacity and reduced cooking loss compared with the results for other ageing periods. Increasing ageing time was also associated with an increase in total free amino acids in both muscles, together with changes in microbial counts [62].

The quality characteristics of goat meat during the *post-mortem* period are also influenced by animal-related factors. Baksh et al. [5] reported that meat from adult goats had a higher proportion of type I muscle fibers, ticker perimysium, and WBSF than did young goats, with these differences remaining evident after 21 days of *post-mortem* storage. These characteristics may contribute to the greater toughness observed in meat from adult goats and are therefore relevant when considering ageing as a strategy for improving the quality of meat from older animals.

Pre-slaughter factors may also influence the subsequent quality characteristics of goat meat. Suliman et al. [65] reported that pre-slaughter treatment affected pH, total bacteria count, e.g., that of Enterobacteriaceae and *Clostridium* spp., and was associated with increased shear force, hardness and chewiness [65].

### 6.1. Color

In red meats such as beef, goat, and pork, color is a key factor influencing consumers purchasing decisions, with the intensity of red widely recognized as an indicator of freshness [87,88]. The stability of the red color is considered the most important factor in the perception of freshness and shelf life [89]. In addition to its aesthetic and commercial relevance, color also has physiological and biochemical significance, providing indirect information on key parameters such as oxygen availability, myoglobin content, and muscle pH [66]. This characteristic results primarily from the chemical state of myoglobin, whose oxidation to metmyoglobin leads to a loss of redness and consequently, product rejection [89].

On retail shelves, consumers perception of color is particularly sensitive to three instrumental dimensions: paleness or lightness (L*), redness intensity (a*) and the tendency towards brownish tones, as reflected mainly in b* [90]. Lightness is associated with both myoglobin concentration and the way light scatters within the muscle structure. At the same time, a* and b* depend on the concentration of this pigment and its chemical state [90]. Color stability depends mainly on the chemical state of myoglobin and oxidative processes, which directly influence redness, lightness and yellowing [91]. During ageing, the oxidation of myoglobin into metmyoglobin and lipid peroxidation are among the most relevant factors contributing to color loss [91]. Objective evaluation uses the CIE Lab* system, where L* indicates brightness or paleness, a* quantifies the intensity of the red component, and b* indicates shifts towards yellowish or brownish tones [59,90].

Stress associated with pre-slaughter handling also plays a significant role in determining meat color by triggering physiological and biochemical changes in the muscle. Excessive stress can lead to elevated post-mortem glycolysis, accelerated pH decline, and higher muscle temperatures, compromising the functionality of muscle proteins and their water-holding capacity, thereby directly impacting meat color [65].

In wet ageing, a progressive increase in L* values has been observed, reflecting a lighter appearance of the meat as ageing time increases [92]. This behavior has been associated with myoglobin oxidation [93], as muscles with a higher content of this pigment tend to have lower L* values [62] (i.e., a darker appearance) [65]. Wet ageing under limited-oxygen conditions promotes myoglobin oxidation and may contribute to variations in lightness during storage, in close interaction with myoglobin content, and in the dynamics of oxidative processes. In addition, the color of meat depends on the rate of oxygen consumption and the meat’s metmyoglobin reduction capacity, both of which influence metmyoglobin formation [93].

From the consumers point of view, higher b* values tend to be positively associated with acceptance. At the same time, reduced redness is often interpreted as a sign of loss of freshness, highlighting the need for effective strategies to preserve the visual appearance of meat throughout its shelf life [91]. The color of goat meat is modulated by several factors and their interactions, notably the lower intramuscular fat content in goat carcasses, which is associated with reduced L* values and higher a* values compared to those of lamb meat [10]. In the specific context of ageing, ref. [27] reported that, except for L* values, no other colorimetric parameters, including a* and b*, showed significant differences, depending on the time and ageing technique used (Table 1). The same authors [27] observed a significant decrease in L* values from week 2 to week 4 in meat subjected to dry ageing in bags, with values lower than those recorded in wet-aged meat. These observations, obtained on freshly cut surfaces, suggest greater susceptibility to surface darkening using the dry-bag method. However, overall color stability was preserved in internal portions of the muscle [27]. In addition, Bakhsh et al. [5] reported that post-mortem storage significantly increased the L* value, with variable patterns in a* and a decrease in b* values [5]. At the same time, adult goats exhibited higher a* values during the early post-mortem period than did young goats, which was attributed to the higher proportion of type I fibers. Packaging methods play a decisive role in color preservation. The absence or low availability of oxygen, as occurs in vacuum packaging or in modified atmospheres with a predominance of N_2_ and CO_2_, reduces lipid oxidation and delays myoglobin oxidation, thus favoring the maintenance of color stability throughout storage. On the other hand, high concentrations of O_2_ in the modified atmosphere, despite initially intensifying redness, accelerate oxidative processes that compromise the product’s quality and shelf life [89].

### 6.2. pH

The pH is a key indicator of goat meat quality, as it influences color, texture, water-holding capacity and microbiological stability during refrigerated storage and ageing [87]. Its evolution after slaughter is primarily determined by the extent of *post-mortem* glucose and is influenced by the animal’s physiological condition and pre-slaughter stress [94]. Goats may be particularly susceptible to stress, which may contribute to higher ultimate values, generally ranging from 5.78–6.3 [95,96], whereas values between 5.4 and 5.8 are commonly considered indicative of normal-quality kid meat [97]. Moreover, pH tends to increase with slaughter age, with adult goats showing higher values than young animals, and this has been associated with differences in muscle fiber composition and *post-mortem* acidification [96]. Because pH affects protein functionality, it also contributes to differences in water-holding capacity, color, appearance and shelf life [6].

During ageing, pH evolution in goat meat varies according to animal age, muscle, ageing method and storage duration. Bakhsh et al. [5] found no significant differences in pH between meat from adult and young goats during the first 14 days of wet ageing, whereas adult goats showed significantly higher values on day 21 (Table 1). These differences were attributed to metabolic characteristics associated with animal age and carcass conformation [5]. In the literature, pH values above 6.0 have been associated with DFD characteristics and reduced shelf life, whereas an excessively rapid decline to values below approximately 5.4 has been associated with PSE characteristics [89,97]. Under normal conditions, *post-mortem* glycolysis progressively reduces muscle pH to approximately 5.4–5.8, with the rate and extent of this decline influencing subsequent physicochemical characteristics [89,94].

Forte et al. [27] reported that ageing time was the only factor significantly affecting pH among the rheological and colorimetric parameters evaluated in goat meat. A decrease in pH was observed between week 0 and week 1 in both dry- and wet-aged meat [27]. In contrast, other studies have reported increases in pH during prolonged ageing. In *Longissimus lumborum* subjected to wet ageing, pH increased significantly after 15 days compared with earlier ageing periods, reaching 6.21 [62]. Similarly, pH values of approximately 6.6 after 14 days and 6.7 after 46 days of ageing have been reported in other studies [57,65]. These differences may reflect variations in ageing conditions, muscle type and ageing duration.

In beef, changes in pH during ageing also vary according to the ageing system and method, as well as the storage conditions. Dry ageing may be accompanied by a slight increase associated with the accumulation of nitrogenous compounds, whereas pH during wet ageing tends to remain relatively stable or may increase slightly, depending on microbial and fermentative activity [55].

### 6.3. Flavor

Meat ageing promotes biochemical and microbial changes that modify the concentration and composition of compounds contributing to flavor. Dry ageing generally produces a more intense and complex sensory profile than does wet ageing, with descriptions including buttery, nutty, earthy, roasted and producing dried-fruit notes, whereas wet-aged meat is more frequently associated with metallic, acidic or bloody characteristics [49]. However, these differences are not consistently observed across studies, indicating that flavor development depends on ageing conditions and duration, raw material, and consumer familiarity with the resulting sensory profiles [49].

Flavor development results from the interaction of proteolysis, lipolysis, carbohydrate degradation, nucleotide metabolism, lipid oxidation and microbial activity [47,49,98]. Proteolysis contributes to the accumulation of peptides and free amino acids, which may directly influence taste and provide substrates for Maillard and Strecker reactions during cooking [47,49,98]. In parallel, the degradation of ribonucleotides contributes to changes in umami and bitter taste, with a decrease in inosine monophosphate (IMP) and guanosine monophosphate (GMP), compounds that enhance the perception of umami flavor, followed by the formation of inosine and hypoxanthine during subsequent degradation [47]. Lipolysis releases free fatty acids that participate in the formation of volatile compounds during cooking, while controlled lipid oxidation generates aldehydes, ketones, alcohols and other compounds contributing to the characteristic aroma of aged meat [47,49,98].

In dry-aged meat, surface microorganisms may further contribute to flavor development. Molds and yeasts, such as *Thamnidium*, *Penicillium*, and *Debaryomyces*, possess proteolytic and lipolytic activities that can modify the composition of proteins and lipids and contribute to the formation of characteristic mushroom, nutty, and spicy notes [47]. These microbial effects, together with the greater exposure of the meat surface to oxygen during dry ageing, help to explain the more pronounced sensory profile generally associated with this method.

Ageing duration is also an important determinant of flavor evolution. Characteristic aged meat notes may become apparent after approximately 14 days and increase with prolonged ageing, although excessive ageing can favor undesirable aromas associated with excessive microbial activity and oxidative processes, which may contribute to the development of undesirable off-odors [49,98]. In goat meat, Forte et al. [27] observed that meat flavor increased during the first weeks of dry ageing, reached a maximum around the second week, and subsequently declined through the fourth and fifth weeks. In wet-aged meat, meaty flavor remained comparatively stable and increased only at later stages [27]. These findings indicate that flavor development in goat meat is strongly dependent on the ageing system and the duration of the process.

The specific composition of goat meat may further influence flavor development. Its relatively high proportion of unsaturated fatty acids provides substrates for oxidation and the subsequent formation of volatile compounds, potentially contributing to characteristic sensory attributes [49].

### 6.4. Tenderness

Tenderness, a parameter of great variability, is one of the most important attributes for consumer acceptance and is influenced by both intrinsic muscle characteristics and *post-mortem* changes occurring during ageing [53,88]. The initial structural characteristics of the muscle determine what is known as “background resistance”, which is mainly determined by collagen concentration and sarcomere length at the onset of *rigor mortis* [99]. Although collagen concentration remains essentially unchanged during *post-mortem* storage, sarcomere shortening during the development of *rigor mortis* directly affects the initial hardness of the meat, subsequently influencing its response to tenderizing processes. This initial resistance interacts with the progressive softening resulting from *post-mortem* proteolysis, mediated mainly by the calpain–calpastatin system, with the final tenderness of the meat being determined by the balance between these two mechanisms, whose relative contribution may vary, depending on the type of muscle [99].

In the *longissimus thoracis et lumborum* muscle of goats, the evolution of tenderness during ageing highlights the importance of time-dependent tenderization processes [65]. An experimental study has shown that the decrease in shear force observed during ageing is associated with the progressive activation of the calpain–calpastatin system, which promotes the degradation of structural proteins and contributes to increased meat tenderness [65]. Although differences in hardness and chewability can be observed between samples in the early stages of ageing, these effects tend to diminish with the *post-mortem* passage of time, indicating that ageing acts as a compensatory mechanism that reduces initial variations in muscle resistance [65]. In goat meat, parameters such as elasticity and cohesion generally remain stable throughout ageing, suggesting that improvements in tenderness are mainly associated with reductions in cutting resistance and hardness, rather than overall changes in muscle tissue structure [65]. The objective evaluation of meat tenderness is often performed using instrumental methods, such as the Warner–Bratzler shear force test (WBSF) and texture profile analysis (TPA), which are widely used as indicators of meat tenderness. These methods quantify the force and work required to cut muscle fibers at a controlled speed, allowing a reproducible assessment of the mechanical resistance of meat [100].

Several studies have shown that, although dry ageing can promote meat tenderness by intensifying the proteolysis of myofibrillar proteins and the consequent degradation of muscle structure [101], high levels of dehydration throughout the process can counteract this effect. Pearce et al. [34] and Wang et al. [100] reported that excessive water loss associated with dry ageing tends to increase cutting resistance, as reflected by higher WBSF values, leading to a lower perception of tenderness. From an oxidative point of view, the more intense dehydration observed in dry-ageing systems can, on the one hand, enhance proteolytic action and improve tenderness in the early stages, but on the other hand, favor a subsequent increase in WBSF values as the process progresses due to greater water loss and the intensification of lipid and protein oxidation reactions [27]. Protein oxidation is associated with cross-link formation between structural proteins and reduced proteolysis, which together increase hardness and further limit *post-mortem* tenderization [27,102].

In goat meat, the lipid profile rich in polyunsaturated fatty acids makes the muscle particularly susceptible to oxidation, with faster increases in TBARS, lipid hydroperoxides, and protein carbonyls under conditions of greater oxygen exposure, such as the dry-ageing process. These phenomena simultaneously lead to the formation of desirable volatile compounds (aldehydes, ketones, aromatic hydrocarbons) in the early stages and in more advanced stages, to the appearance of compounds associated with unpleasant flavors and odors, which coincides with the degradation of overall sensory quality and the perception of less tender texture [27]. In addition to the type and duration of ageing, the tenderness of goat meat is also strongly influenced by the age at slaughter. Regarding slaughter age, young animals exhibited consistently lower WBSF values and higher myofibrillar fragmentation index levels than those of adult animals, indicating more tender meat. This effect is mainly attributed to lower connective tissue cross-linking in young animals, since older animals have more cross-links in the perimysium, contributing to greater structural toughness [5].

Studies also reported significant decrease in WBSF values over 21 days of refrigerated storage, indicating a progressive increase in tenderness, an effect attributed mainly to the action of endogenous proteinases that degrade myofibrillar proteins during *post-mortem* ageing. At the same time, the increase in sarcomere length and the myofibrillar fragmentation index over time reinforces the central role of proteolysis in improving tenderness [5].

## 7. Future Perspectives

Important knowledge gaps remain regarding ageing technologies for goat meat. Future studies should establish ageing conditions specifically tailored to this species, including temperature, relative humidity, air velocity, ageing duration and packaging systems. Greater attention should also be paid to differences among muscle types and slaughter ages, as these factors may influence proteolytic activity, lipid oxidation, moisture loss, tenderness, flavor development and overall stability. Comparative studies involving dry ageing, wet ageing, and dry ageing in bags would be particularly valuable for determining how each system affects product quality, yield and acceptability.

Future research should combine microbiological, physicochemical, sensory and molecular approaches to establish reliable safety criteria and distinguish desirable surface microbiota. In parallel, consumer-oriented studies including items such as consumer’s perceptions of flavor, tenderness, and appearance, as well as their willingness to purchase and perceived value, are needed to assess the acceptability of aged goat meat.

Further studies should assess processing costs, weight and trimmings losses, equipment and infrastructure requirements, product yield and potential market prices. This information will be essential for determining whether dry ageing and dry ageing in bags can be implemented on a commercially viable scale.

## 8. Conclusions

The available evidence indicates that ageing can improve tenderness and influence key physicochemical and sensory attributes, including color, water-holding capacity and flavor. Dry ageing may additionally promote the development of distinctive sensory characteristics, whereas wet ageing can provide quality improvements while limiting moisture and trimming losses. Ageing processes represent a promising technological strategy for enhancing the quality attributes and commercial value of goat meat, although their effectiveness is strongly dependent on ageing conditions, muscle-specific characteristics, and animal age.

## Figures and Tables

**Table 1:** Physicochemical characterization (pH and color) of goat meat subjected to different ageing processes.

Ageing Temperature	Aged Muscle	Type of Ageing	Age of Animals	Ageing Time	pH	Color			Reference
						L*	a*	b*	
2 °C	Hind limbs	Wet ageing	7 years	1 week	5.58	38.14	18.71	1.65	[27]
				2 weeks	5.48	40.62	18.53	1.37	
				3 weeks	5.61	35.34	17.32	0.78	
				4 weeks	5.52	35.50	17.23	0.85	
				5 weeks	5.33	34.69	18.12	0.05	
		Dry ageing		1 week	5.64	35.96	18.41	0.24	
				2 weeks	5.59	39.93	18.03	0.34	
				3 weeks	5.64	37.95	18.76	0.28	
				4 weeks	5.70	40.52	17.81	1.73	
				5 weeks	5.74	38.25	17.85	0.45	
4 °C	*Longissimus thoracis*	Wet ageing	9 months	7 days	5.59	44.78	21.44	4.36	[5]
			18 months		5.63	44.56	22.02	4.31	
			9 months	14 days	5.61	44.88	20.62	4.31	
			18 months		5.64	44.28	21.08	4.36	
			9 months	21 days	5.58	45.40	20.40	4.17	
			18 months		5.67	45.28	20.54	4.47	
2 ± 2 °C	*Longissimus thoracis et lumborum*	Dry ageing in bags	7–8 years	46 days	6.7	34.8	14.5	5.1	[57]
4 °C	*Longissimus lumborum*	Wet ageing	16 months	5 days	5.88	38.07	20.46	9.86	[62]
	*Biceps femoris*				6.07	37.84	21.28	9.00	
	*Longissimus lumborum*			10 days	5.96	38.14	21.68	9.57	
	*Biceps femoris*				6.04	36.8	20.02	7.10	
	*Longissimus lumborum*			15 days	6.21	36.70	20.48	9.80	
	*Biceps femoris*				5.93	36.13	25.03	11.07	
4 ± 1 °C	*Longissimus thoracis et lumborum*	Wet ageing	10 months	1 day	5.90	44.4	15.68	14.26	[65]
				7 days	5.88	34.34	11.83	11.84	
				14 days	6.90	30.02	10.70	8.99	

**Table 2:** Comparative summary of goat meat-ageing studies according to ageing conditions and quality outcomes.

Ageing Method	Temperature	Muscle	Ageing Duration	Tenderness (WBSF, kgf)	Sensory Response	Microbiological Quality (Log CFU/g)	Reference
Wet ageing	4 ± 1 °C	LTL	3 days	13.66–15.71	--	--	[66]
			7 days	8.92–10.75			
		QF	3 days	8.51–19.43			
			7 days	6.99–22.45			
Dry ageing	2 °C	HL	1 week	5.05	7.31	--	[27]
			2 weeks	4.85	8.91		
			3 weeks	5.02	7.28		
			4 weeks	5.12	5.31		
			5 weeks	5.09	4.12		
Wet ageing			1 week	5.44	6.58		
			2 weeks	5.15	7.08		
			3 weeks	5.11	6.89		
			4 weeks	5.22	6.93		
			5 weeks	5.12	6.69		
Wet ageing	2 °C	LL	1 day	3.15–3.48	--	--	[11]
			3 days	2.62–3.14			
			6 days	2.89–3.14			
Wet ageing	4 °C	LL	5 days	2.16	--	TBC: 3.72	[62]
						C: 3.30	
			10 days	2.23	--	TBC: 5.94	
						C: 5.28	
			15 days	3.19	--	TBC: 6.96	
						C: 6.40	
		BF	5 days	3.58	--	TBC: 3.28	
						C: 2.28	
			10 days	4.17	--	TBC: 5.79	
						C: 4.15	
			15 days	4.03	--	TBC: 7.24	
						C: 5.12	

Notes: BF (*biceps femoris*); C (coliform); HL (hind limbs); LL (*longissimus lumborum*); LTL (*longissimus thoracis et lumborum*); QF (*quadriceps femoris*); TBC (total bacteria count); WBSF (Warner–Bratzler Shear Force); -- (Not reported). In the sensory evaluation, the overall liking is given on a scale of 1 to 10.

## Data Availability

No new data were created or analyzed in this study. Data sharing is not applicable to this article.

## Abbreviations

The following abbreviations are used in this manuscript:

DFD	Dark, Firm and Dry
GMP	Guanosine Monophosphate
HACCP	Hazard Analysis and Critical Control Point
IMP	Inosine Monophosphate
LAB	Lactic Acid Bacteria
PSE	Pale, Soft and Exudative
TBARS	Thiobarbituric Acid Reactive Substances
TPA	Texture Profile Analysis
CFU	Colony-Forming Unit
WBSF	Warner–Bratzler Shear Force
